# Circular RNAs: biogenesis, expression and their potential roles in reproduction

**DOI:** 10.1186/s13048-018-0381-4

**Published:** 2018-01-17

**Authors:** Guobo Quan, Julang Li

**Affiliations:** 1 Yunnan Animal Science and Veterinary Institute, Jindian, Panlong county, Kunming, Yunnan province 650224 China; 2 Department of Animal Biosciences, University of Guelph, 50 Stone Road East, Building #70, Guelph, ON N1G 2W1 Canada; 3 College of Life Science and Engineering, Foshan University, Foshan, Guangdong province China

**Keywords:** Circular RNA, Reproduction, Gene regulation, miRNA, lncRNA

## Abstract

Unlike other non-coding RNAs (ncRNAs), circular RNA (circRNA) is generally presented as a covalently linked circle lacking both a 5′ cap and a 3′ tail. circRNAs were thought to be spliced intermediates, byproducts, or products of abnormal RNA splicing events. However, the high-throughput sequencing technology coupled with bioinformatics has recently uncovered thousands of endogenous circRNAs in cells of many different species. These circRNAs show various features, such as abundant expression, evolutionary conservation, cell- or tissue-specific expression, and a higher resistance to degradation caused by exonuclease or ribonuclease (RNase), suggesting their potentially biological significance. However, the function of these circRNAs, their mechanism of action, and the regulation of their biogenesis and degradation remains largely unclear. The current research and findings of circRNA in the context of reproduction will be reviewed. Additionally, the perspectives of circRNAs in the field will be discussed.

## Background

As a subclass of ncRNAs, circRNAs are not novel because of their natural existence in some low grade organisms or plants. A typical circRNA class has been detected in some viruses, such as the hepatitis D virus [1]. HDV was initially discovered in some Italian patients infected with the hepatitis B virus [2]. Its structure was proven to be a covalently-linked circle based on its biomedical and electron microscopic analysis [1]. In addition, some plant viroids are presented as a covalently bound circular RNA molecule [3, 4]. The existence of circRNAs in mammalian cells, however, was heavily debated. An important finding was reported in 1979 when some interesting RNAs with a circular structure in the eukaryotic cytoplasm were identified using an electron microscope [5]. However, due to their low abundance and uncommon features, these RNAs were only regarded as byproducts of abnormal splicing and did not receive much attention at the time. Later in 1991, when studying the expression of a tumor suppressor gene, some circular transcripts were detected in human cells although the significance of these circRNAs remained unclear [6].

With the advancement of high-throughput sequencing technologies and bioinformatics, an important breakthrough discovery in circRNA research appeared in 2012. In a study initially designed to screen genomic rearrangements associated with cancer, the global expression of circRNAs was accidentally discovered in the RNA sequencing (RNA-seq) data of human pediatric acute lymphoblastic leukemia samples. Additionally, the existence of circRNAs was further confirmed in both healthy and cancerous cells [7]. Based on the RNA-seq data, it is estimated that approximately 100,000 circRNAs are expressed in humans [7]. The higher abundance and diversity of circRNA expression may be associated with the alternative splicing of RNA transcripts [8–11]. Additionally, most circRNAs are presented in the cytoplasm, which is consistent with their regulatory role in the post-transcriptional function [7, 12, 13]. However, the newly discovered circular intron circRNAs (ciRNAs) or exon–intron circRNAs (EIciRNAs) are primarily located in the nucleus, which may correlate with their regulatory roles in their parental gene transcription [14, 15].

Although thousands of circRNAs have now been discovered, the ability to utilize the vast amount of circRNAs in the database poses a great challenge. At present, some features of circRNAs, such as high abundance, diverse structures, high resistance to degradation by exonuclease or RNase, cell- or tissue-specific expression, and highly evolutionary conservation, have been confirmed [11, 16, 17]. However, the mechanisms regulating their biogenesis, function, degradation, and cellular localization remain largely unclear [18, 19].

In the field of reproduction, investigations about circRNAs in the ovary, testis, and placenta have been reported. However, these reports mainly focus on circRNA screening and expression pattern [20–22]. Some of the studies suggested that circRNA may be engaged in epigenetic regulation and embryonic development [20, 21, 23]. These studies shed light on the role of circRNAs in the reproductive system.

### The putative mechanism regulating circRNA biogenesis

Most circRNAs consist of protein-coding exons and are covalently circulated by canonical splicing sites. The canonical spliceosomal splicing mechanism is thus believed to be engaged in the regulation of circRNA biogenesis [11, 16, 24, 25]. A support for the involvement of a canonical spliceosomal splicing mechanism in circRNA biogenesis is that isoginkgetin, a splicing inhibitor, simultaneously blocks the formation of both linear and circular RNAs [11, 24]. Additionally, the induced mutation of canonical splicing sites interfered with exon circularization, subsequently prohibiting circRNA biogenesis [16, 26].

The current mainstream opinions tend to approve the regulatory roles of the backsplicing mechanism during circRNA biogenesis [7, 13, 19, 27]. The backsplicing mechanism is different from the canonical linear splicing mechanism based on the sequence of its splicing donors and acceptors [16, 25]. In canonical splicing, an upstream (5′) splice donor site is linked to a downstream (3′) splice acceptor site. Coupled with other transcriptional and post-transcriptional processes including 5′ capping and 3′ polyadenylation, canonical splicing produce a linear RNA transcript with 5′ to 3′ polarity [28]. By contrast, in backsplicing, a downstream (3′) splice donor site reversely accesses an upstream (5′) splice acceptor site, consequently forming a covalently linked circRNA and a linear RNA with skipped exons [28]. However, circRNA biogenesis regulated by the backsplicing mechanism is still influenced by both canonical splicing signals and spliceosomal machinery [11, 24]. Since most of the highly expressed circRNAs are generally derived from internal exons of pre-mRNAs, backsplicing is generally coupled with canonical splicing [28].

Meanwhile, there is a competitive relationship between the canonical linear splicing and circRNA biogenesis. The mechanism regulating this competition is tissue-specific and highly conserved between flies and humans [24]. Moreover, circRNA generation has been shown to be influenced by the transcriptional elongation velocity. In a recent study, 4-thiouridine labeling was used to identify newly produced circRNAs, suggesting a faster transcribing velocity (2.9 kb/min) when compared to non-circRNA transcriptions (2.29 kb/min) [29]. Therefore, the backsplicing efficiency is positively influenced by the elongation velocity of RNA polymerase II [29]. For example, a mutation of a large subunit of the RNA polymerase II can greatly reduce the elongation velocity of RNA polymerase II, subsequently leading to a lower backsplicing efficiency in flies or mammals [30–32]. Additionally, according to the ratio between linear and circular RNAs, the circRNA expression level in flies with the mutation was significantly reduced [24], further supporting the effects of canonical splicing on circRNA formation.

circRNA biogenesis can be promoted by inverted repeats existing on both sides of exons. The base-pairing formation between these inverted repeats contributes to RNA circularization due to the induced spatial reduction among the splice signals involved in backsplicing [13, 15, 33]. Additionally, the existence of inverted ALU repeat elements (IAREs) can promote exon circularization [13]. In humans, IAREs, as critical components of complementary sequences and flanking introns, play an important role during exon circularization [34].

Some RNA binding proteins (RBPs), such as muscleblind (MBL) and quaking (QKI), are believed to promote circRNA biogenesis. MBL strongly and specifically binds to the circRNA derived from its own RNA. Owing to the regulation by MBL, a circRNA derived from the second exon circulation of Mbl pre-mRNA (circMbl) can be formed, which relies on the presence of conservative binding sites for MBL in the introns flanking the circularized exons [24, 35]. In addition, the exogenous expression of fly MBL enhances circRNA biogenesis from endogenous fly and human muscleblind transcripts. Down-regulation of MBL in mammalian cell culture or fly neural tissue significantly reduced the expression level of circMbl [24]. QKI, another important RBP, can also positively regulate circRNA formation. QKI contributes to circRNA biogenesis during the epithelial-to-mesenchymal transition (EMT) in human immortalized mammary epithelial cells [26]. The evidence that the knockdown of QKI blocks the expression of circRNAs related to the EMT further verifies the positive roles of QKI during circRNA biogenesis [26]. It should be noted that the regulatory roles of QKI require the involvement of putative binding sites in the flanking introns of circularized exons [26]. However, whether MBL or QKI can form base-pairing between two introns during circRNA biogenesis still lacks direct evidence.

### The circRNA subclasses and their biogenesis

**Fig. 1:**
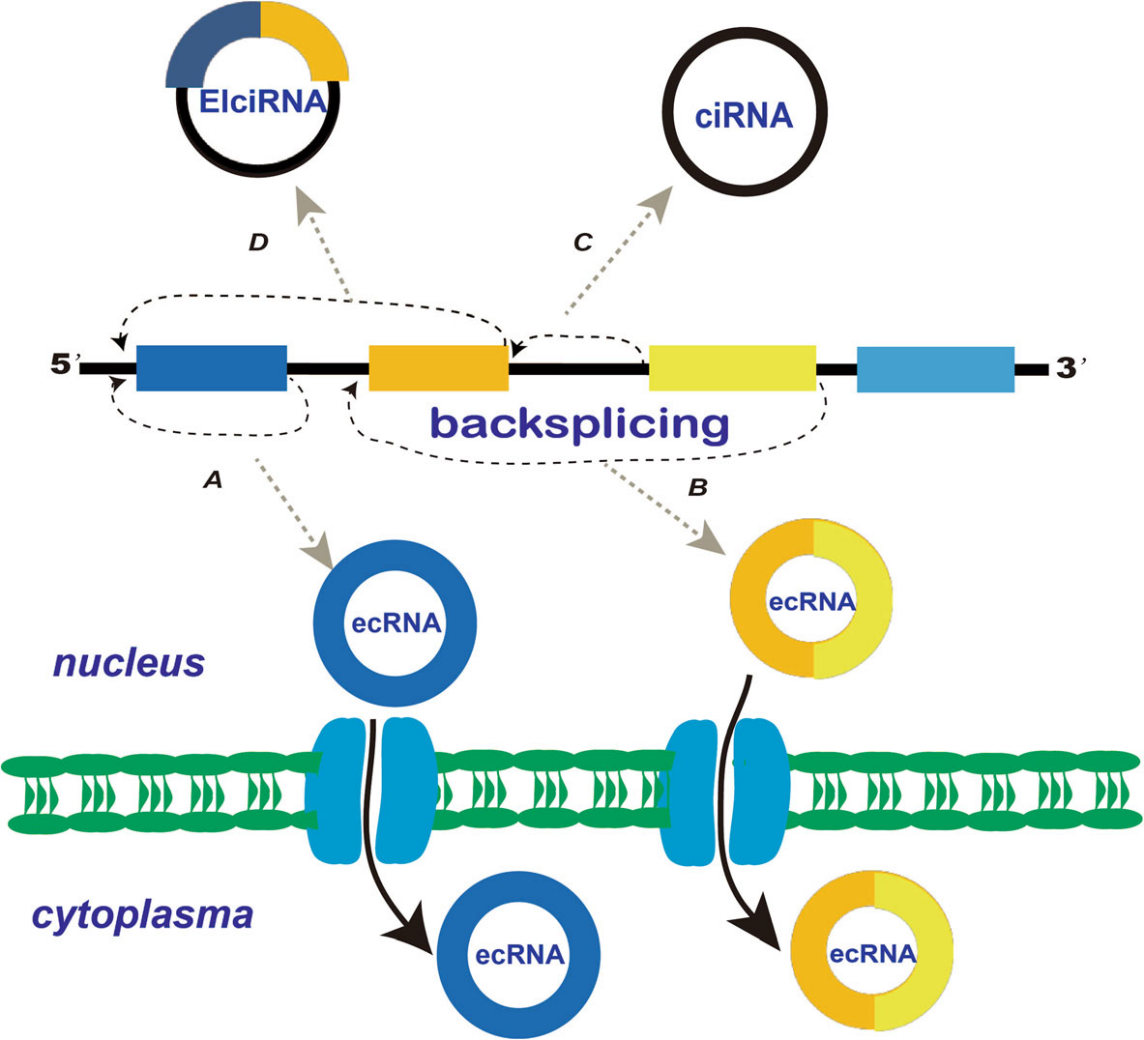
The biogenesis of circRNAs. In the figure, exons are represented by different coloured rectangles and introns are represented by black lines. Exon-derived circRNA (ecRNA) only consists of exons (a and b). circular intronic RNA (ciRNA) contains only introns (c). In exon–intron circRNA (EIciRNA), an intron is inserted between two exons (d). The pathway through which mature ecRNAs migrate into the cytoplasm remains unclear. In this figure, circRNAs are postulated to pass through the nucleus membrane via a nuclear pore complex

As illustrated in Fig. 1, circRNAs consist of three subclasses based on their generating pathways. The most popular circRNA is the exon-derived circRNA (ecRNA), containing only exons and completely lacking introns [16]. Two mechanisms, including both exon skipping and direct backsplicing, have been proposed to explain ecRNA biogenesis [13, 25, 36]. Exon skipping, as a form of RNA splicing during mRNA biogenesis, may be associated with ecRNA formation. During exon skipping, the downstream exon rotates and leaps one or serveral exons to link the upstream exon, consequently producing a exon-skipped and functional mRNA. Meanwhile, the leaped exons form a lariat precursor containing exons and inrons, finally forming a circRNA after removal of introns [13, 37, 38]. By contrast, the direct backsplicing first generates alternatively spliced RNA and a lariat intermediate regulated by the intron-pairing mechanism. The introns in the lariat are then removed by the canonical splicing process [7, 21, 39–41]. Recent evidence indicates that direct backsplicing, not exon skipping, may be the primary mechanism regulating ecRNA formation [42]. After their biogenesis, ecRNAs must migrate into the cytoplasm to play their regulatory roles. However, the mechanism regulating the migration of mature ecRNAs into the cytoplasm currently remains unclear. The linear counterparts of circRNAs, such as mRNAs or long non-coding RNA (lncRNAs), can penetrate the nuclear membrane through the nuclear pore complex. It was suggested that ecRNA export may be regulated by a mechanism similar to the regulatory mechanism of linear RNA migration [18]. The degradation pathway of circRNAs is another unanswered question. The expression level of circRNA is dynamically modulated by the balance between biogenesis and degradation of circRNAs. circRNAs may be degraded via a mechanism triggered by short interfering RNAs [13]. A recent study found that circRNAs may be cleared by extracellular vesicles or microvesicle release in mammalian cells [43]. However, this conclusion is based on an in vitro study. Whether circRNAs are degraded in vivo by a similar mechanism still needs further elucidation.

In 2013, a novel type of circRNAs consisting of only introns, referred to as ciRNA, was discovered in human cells. It is mainly presented in the nucleus and is involved in the transcriptional regulation of its parental genes [14, 15]. ciRNA biogenesis requires a consensus motif consisting of both a 7 nt GU-rich element near the 5′ splice site and an 11 nt C-rich element near the branchpoint site. This motif may be specifically engaged in ciRNA formation because it is not enriched in regular introns or other types of circRNAs [44]. ciRNA biogenesis is regulated by a eukaryotic spliceosome-mediated splicing mechanism. ciRNAs are circular introns which are circularized at the branchpoint 2′–5′ linkage and degraded from the 3′ end up to the branchpoint. Therefore, ciRNAs are highly stable, due to their resistance to debranching and degradation [15, 45].

Recently, a more interesting EIciRNA in which one intron is inserted between two exons was discovered (illustrated in Fig. 1). Similar to ciRNA, EIciRNAs may also regulate their parental gene transcription in the nucleus [14]. However, the mechanism regulating EIciRNA biogenesis is unclear.

### The putative functions of circRNAs

**Fig. 2:**
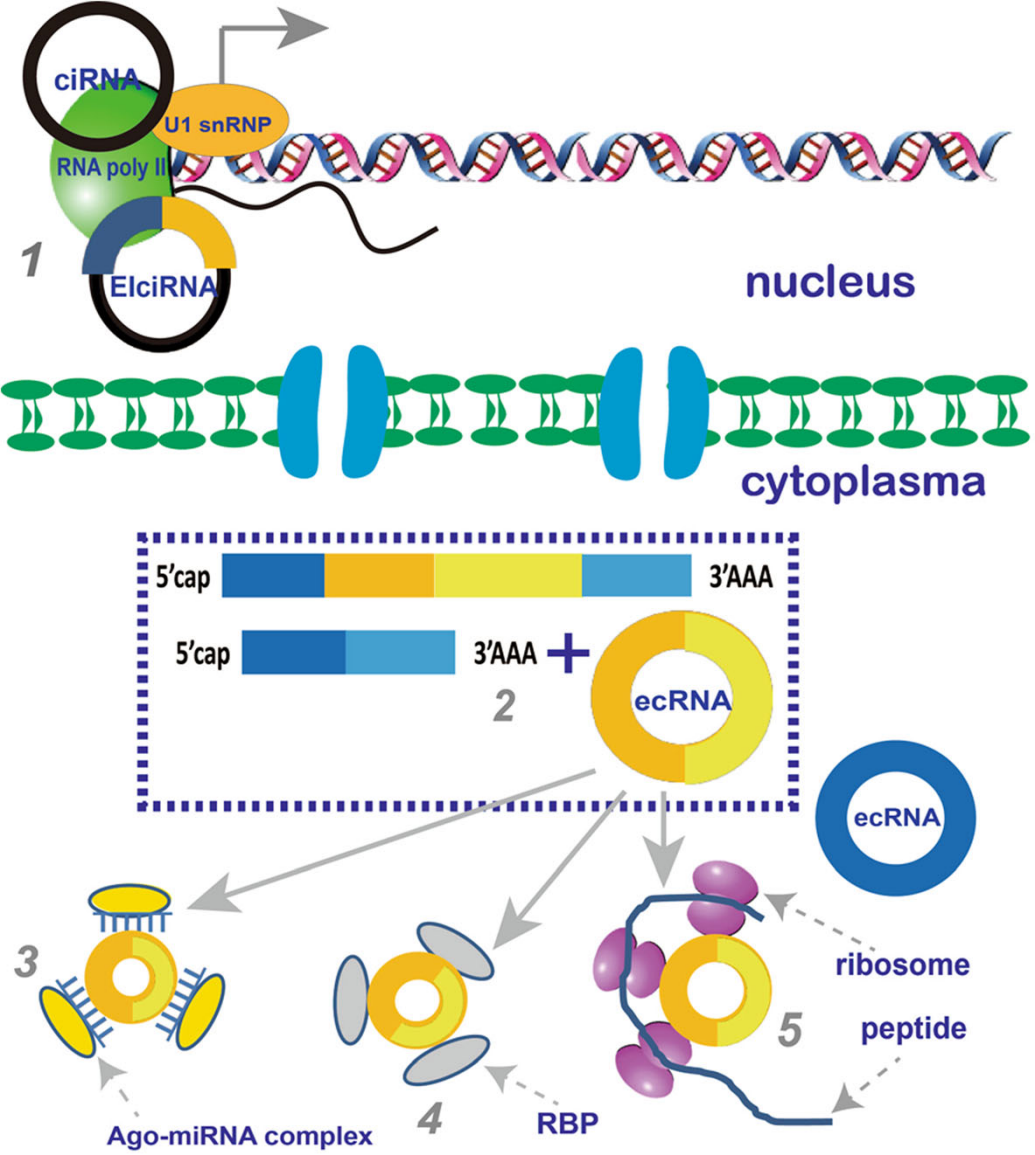
The putative function of circRNAs. In this figure, exons are represented by different coloured rectangles and introns are represented by black lines. Five potentially biological functions of circRNAs are suggested. 1) To promote transcription of their parental genes: the ciRNAs and EIciRNAs are located in the nucleus and involved in their parental gene expression. 2) mRNA trap: the biogenesis of circRNAs is generally accompanied with transcription of their parental genes. Therefore, circRNAs may competitively influence the biogenesis and processing of mRNA. 3) miRNA sponge: some circRNAs have conserved binding sites for miRNAs. Through competitively binding with miRNAs, these circRNAs can block the binding between miRNAs and their target mRNAs, subsequently prohibiting the repressive effects of miRNAs on the target protein translation. 4) RNA binding protein (RBP) sponge: to regulate the function of RBPs, some circRNAs can interact with RBPs, such as Argonaute (Ago), polymerase II, muscleblind, etc. 5) to encode protein: some circRNAs contain internal ribosome entry site which can bind with ribosomes (shown by two tightly-attached ellipses). Therefore, these circRNAs may have an ability to encode proteins

circRNAs are important modulators involved in the transcriptional and post-transcriptional regulation of gene expression. As the linear counterpart of circRNAs, lncRNAs can fold into complex secondary or higher structures to provide more potential and diversity for both protein and target recognition [46, 47]. Therefore, the functional patterns of lncRNA are highly complicated. On the contrary, the structure of circRNA is circular and lacks complex spatial folding. The functional roles of circRNAs are therefore mainly determined by their base sequence. The putatively functional roles of circRNAs are summarized in Fig. 2.

First, circRNAs can function as a miRNA sponge to regulate the function of miRNAs [19, 27]. Some circRNAs, such as circRNA for miRNA-7 (ciRS-7) or Sry circRNA (circSry), contain some conserved binding sites for miRNAs. Based on the miRNA sponge theory, miRNA, in a form of Argonaute-miRNA complex, can bind to circRNAs. Therefore, circRNAs may function as competitively endogenous RNAs to block the miRNA-mediated endo-cleavage pathway [45]. Through competitively binding with miRNAs, circRNAs can interfere with the binding procedure between miRNAs and their target mRNAs, consequently weakening the repressive effects of miRNAs on the target protein translation [19, 27]. For instance, ciRS-7 possesses 74 conserved binding sites which can be bound by miR-7. Meanwhile, ciRS-7 can also be bound by Argonaute proteins which can interact with miRNAs [19]. Currently, the coexistence of both ciRS-7 and miR-7 has been affirmed by in vitro microscopy and co-immunoprecipitation experiments [27]. The study of transfecting miR-7 into HeLa cells has revealed that the response of constructed miR-7 targets is more efficient in the cells lacking ciRS-7 expression in comparison with cells expressing ciRS-7 [27]. In addition, miR-7 has shown great potential for applications in cancer therapy [48]. miR-7 can down-regulate some factors associated with cancer signaling pathways, such as epidermal growth factor receptor (EGFR), insulin-receptor substrate-1 (IRS-1), insulin-receptor substrate-2 (IRS-2), rapidly accelerated fibrosarcoma 1 (Raf1), p21-activated kinase 1 (Pak1), activated cdc42 kinase 1 (Ack1), insulinlike growth factor-1 receptor (IGF1R), phosphatidylinositol-4,5-bisphosphate 3-kinase catalytic subunit delta (PIK3CD), and mammalian target of rapamycin (mTOR) [48]. Additionally, E-cadherin can be up-regulated by miR-7, which can target IGF1R [49, 50] and focal adhesion kinase (FAK) [51, 52], causing weakened EMT, reduced anchorage-independent growth, and suppressed metastasis [48]. Therefore, ciRS-7 may play a regulatory role in oncogenesis via its interaction with miR-7.

In a previous study, the overexpression of ciRS-7 resulted in a decrease of the zebrafish midbrain size in zebrafish embryos. Similar phenomena have also been observed after silencing miR-7 via injection of morpholinos to reduce the expression level of miR-7 in zebrafish embryos [19], suggesting that the alteration of ciRS-7 can reversely influence the expression of miR-7. Furthermore, as a natural miRNA sponge for miR-7, ciRS-7 may regulate the expression of miR-7 targeted genes, some of them are known to be associated with Parkinson’s disease or Alzheimer’s disease [53].

Another example is circSry, which contains 16 conserved binding sites and can act as a miRNA sponge to sequester miR-138 [27]. Wang et al. recently discovered a new circRNA-heart-related circRNA (HRCR) which can function as a sponge to repress the activity of miR-223, subsequently leading to a weakened hypertrophic response [54]. These studies suggest that the miRNA sponge may be an important aspect of circRNA function. However, contradictory findings have also suggested that serving as a miRNA sponge may not be a universal role of circRNAs [12, 42, 45]. In order to test the miRNA sponge roles of circRNAs, Militello et al. used the list of circRNAs in the circBase database (http:// www.circbase.org) [55] and screened for potential regions which can be bound by Argonaute [56]. Their results indicated that there were approximately 58,063 circRNAs with Argonaute-bound regions among 92,375 circRNAs in the circBase database. The reason why numerous circRNAs possess Argonaute-bound regions may be due to the fact that circRNAs are mainly derived from protein-coding genes which may have Argonaute-binding sites in their sequences [56]. Several highly-expressed circRNAs were selected to verify their miRNA sponge roles using the RNA-binding protein immunoprecipitation- polymerase chain reaction (RIP-PCR) technology. The results, however, were negative which does not support miRNA sponge as a universal function of circRNAs.

Secondly, circRNAs can interact with some RBPs, such as Argonaute [19, 27], RNA polymerase II [15], or MBL [24]. Some circRNAs may act as a RBP sponge to regulate the function of RBPs [19]. In a study carried out by Guo et al., based on the data collected from 20 RBPs in the high-throughput in vivo crosslinking experiment, the cluster densities of circular exons were slightly higher than those of their neighboring exons, possibly due to the fact that RBPs bound with circRNAs cannot be replaced by the translocating ribosome owing to the lack of translating capability in circRNAs [12]. However, in another study, the bioinformatics analysis of 38 RBPs revealed that the density of circRNAs bound with RBPs was lower in comparison with the coding sequence or 3′ untranslated regions (UTRs) of mRNAs [57]. In another study, in order to verify regulatory interactions between circRNAs and RBPs, circScan was adopted to detect backsplicing reads from cross-linking and immunoprecipitation followed by the high-throughput sequencing [58]. The authors screened 1500 crosslinking-immunprecipitation and high-throughput sequencing (CLIP-seq) datasets, identified approximately 12,540 novel bindings between circRNAs and RBPs in the human genome, and 1090 in the mouse genome. Additionally, these results were further confirmed by the RNA immunoprecipitation quantitative PCR (RIP-qPCR) [58]. It is speculated that the circRNAs and RBPs interaction may be engaged in diverse biological processes.

The third potential function of circRNAs is to act as an mRNA trap. Since circRNAs mainly consist of exons which may also be involved in mRNA biogenesis, circRNA formation may compete with the biogenesis of linear mRNA [45, 59]. However, there is evidence against this hypothesis. A typical example of this is the fmn gene. The mutations of the acceptor site in the fourth or fifth exon of the flavin mononucleotide (fmn) gene can cause the removal of corresponding circRNA. However, these mutations cannot influence the splicing efficiency of linear RNAs [59]. Therefore, whether the mRNA trap is merely a byproduct of circRNA biogenesis or an intended product is still unknown.

As newly discovered circRNAs, EIciRNA and ciRNA have been reported to be involved in the transcriptional regulation of their parental genes [14, 15]. EIciRNAs are generally located at the transcriptional site in the nucleus [14]. For example, either circEIF3J or circPAIP2 exists with its corresponding parental genomic loci and the U1 small nuclear ribonucleoprotein particle (U1 snRNP). EIciRNAs promote the transcription of their parental genes *in cis* with the assistance of the U1 snRNA, which may primarily function via the specific RNA-RNA interaction between U1 snRNA and EIciRNAs. Meanwhile, the down-regulation of EIciRNA expression levels can decrease the expression level of their parental mRNA [14], further suggesting the positive effects of EIciRNAs on the transcription of their parental mRNAs. Similar phenomena are also observed in ciRNAs which have been reported to be involved in the regulation of their parental gene expression [15].

Some circRNAs contain internal ribosome entry sites which can interact with ribosomes, suggesting that they may be translated into proteins [42, 60]. However, earlier works reported that most circRNAs cannot interact with polyribosomes, which leads to the viewpoint that circRNAs translation is less likely [12, 13, 39, 61]. However, as the linear counterparts of circRNAs, lncRNAs encodes some low-conservative peptides, which have been confirmed by ribosome profiling and mass spectrometry [62–64]. Therefore, whether circRNAs can encode proteins still remains a enticing question.

Recently, it was reported that a circRNA with an open reading frame can act as a template to guide protein translation in living cells by the rolling-circle amplification mechanism [65]. More recently, the methylated adenosine, N6-methyladenosine (m^6^A), has been reported to promote efficient initiation of protein translation from circRNAs in human cells [66]. Through the polysome profiling, mass spectrometry and computational analysis, the same group also found that the consensus m^6^A motifs are enriched in circRNAs, suggesting that they may have translation potential [66]. Similarly, Li et al. also confirmed that some natural circRNAs can interact with cap-independent translation factors including eukaryotic initiation factor 3 and m^6^A, suggesting that these circRNAs may have a capability of translating proteins [58]. Based on a recent ribosome footprinting dataset analysis, Pamudurti et al. reported that 37 circRNAs were associated with translating ribosomes. In the same study, it was revealed that a circRNA derived from the muscleblind locus can be translated into a protein in a cap-independent manner. The produced protein has been detected in *Drosophila* head extracts by mass spectrometry [67]. Similarly, Legnini et al. also demonstrated that circ-ZNF609, a circRNA specifically regulating the proliferation of myoblasts, can bind to heavy polysomes, which has been confirmed by the sucrose gradient fractionation analysis, finally being translated to a protein in a splicing-dependent and cap-independent manner [68]. Additionally, after treatment with puromycin, circ-ZNF609 in myoblasts shifted to lighter polysomes in a way similarly to that of the corresponding ZNF609 mRNAs, showing the existence of active translation [68]. Another interesting phenomenon is that the presence of poly-adenosine or poly-thymidine in 3′ UTR blocks circRNA translation, which is different from mRNA [25]. Therefore, more and more new evidence is continually emerging to support the protein encoding function of circRNA.

### circRNA and reproduction

Despite several sporadic reports about the expression and potential biological functions of circRNAs in reproductive organs, the research regarding the functional roles of circRNAs in this field is still at its infancy. However, these early stage studies shed light for further investigation. The current reports about circRNAs in the reproductive system are summarized in Table 1.

**Table 1:** The recent advances about circRNAs related to reproduction

Species	Sample	Detection methods	Special treatment	Number of circRNAs	References
Xenopus tropicalis	Oocytes	RNA-sequencing	rRNA depletion	About 9000 lariat RNAs	Talhouarne and Gall [70]
Mouse	Embryo	Single-cell universal poly (A)-independent RNA sequencing	none	2891 circRNAs	Fan et al. [71]
Human	Embryo	Single-cell universal poly (A)-independent RNA sequencing	RNase H degradation	10,032 circRNAs from 2974 hosting genes	Dang et al. [23]
Human	Placental tissue	Arraystar circRNA microarray technology	none	301 circRNAs with different expression	Qian et al. [21]
Mouse	Spermatogenic cells	RNA sequencing	RNase R Treatment	15,101 circRNAs in spermatogenic cells	Lin et al. [82]
Human	Testis	RNA sequencing	RNase R Treatment	15,996 circRNAs containing 10,792 new circRNAs	Dong et al. [20]
human	granulosa cells	circRNA microarray technology	RNase R Treatment	57 differentially expressed circRNAs	Cheng et al. [77]

### The expression of circRNAs in oocyte and pre-implantation embryo development

Thousands of stable RNAs derived from the introns of the most expressed genes were detected in the cytoplasm and the nucleus of *Xenopus tropicalis* oocytes. These RNAs were named as stable intronic sequence RNAs (sisRNAs) [69, 70]. Approximately 9000 sisRNAs have been identified in the cytoplasm of amphibian oocytes. These RNAs are resistant to the degradation induced by exonucleases and RNase R, due to their lariat structure [70]. Another interesting phenomenon is that the abundance of sisRNAs remained consistent from the germinal vesicle breakdown stage to the blastocyst stage of embryogenesis. Furthermore, the ratio between mRNA and sisRNA did not alter during oocyte and embryo development [70]. The expression pattern of sisRNAs suggests that they may regulate mRNA translation or stability during embryogenesis [70]. However, the functional roles of these sisRNAs in *Xenopus tropicalis* oocytes remain largely unclear. Additionally, Zhang et al. suspected that the biogenesis of sisRNAs may be via a mechanism similar to that of ciRNAs which regulate their parental gene expression [15].

The expression of circRNAs in pre-implantation embryos has only been reported in mice [71] and humans [23] to date. A novel single-cell universal poly (A)-independent RNA sequencing (SUPeR-seq) technology has been used for deep sequencing of mouse pre-implantation embryos [71]. A total of 2891 circRNAs derived from 1316 host genes were detected in mouse oocytes and early embryos (from zygotes to blastocysts). Most circRNAs, with a length of less than 2 kb, were derived from internal exons in the same host gene. Moreover, about 91% of circRNAs consist of multiple exons [71]. Different from what has been reported in amphibians [70], the expression pattern of these circRNAs shows a typically developmental stage-specific feature. Additionally, the gene ontology analytical data of the host genes producing these circRNAs revealed that the circRNAs presented in early embryos of mice are potentially responsible for chromosome organization, cell division, and DNA repair [71].

The same group analyzed the number of circRNAs at each embryonic stage. Approximately 2278 circRNAs were expressed in metaphase II mouse oocytes. After fertilization, the circRNA number in zygotes sharply reduced to 1850. At the four-cell embryo stage, the number of circRNA further reduced to only 1422. However, at the morulae stage, the circRNA number increased to 2799 instead. Again, the circRNA number strikingly decreased to only 779 in the blastocyst stage, suggesting the existence of a circRNA degradation mechanism during the morulae-to-blastocyst transition [71]. The fluctuation of circRNA abundance in mouse oocytes and pre-implantation embryos may be associated with their specific roles at different developmental stages.

Using a similar approach, 10,032 circRNAs derived from 2974 host genes were detected in human pre-implantation embryos [23]. Based on the differential expressed gene analysis, 1554 maternal genes and 851 zygotic genes were identified as host genes [23]. The expression of most circRNAs is developmental stage-specific and dynamically regulated. Many circRNAs are maternally expressed, suggesting their potentially regulatory roles during oogenesis and generation of totipotent zygotes [23].

The circRNAs expressed in human pre-implantation embryos are much more abundant than those presented in mouse embryos, which may be partially due to the increase in intron length during human genome evolution [23]. However, circRNA biogenesis is generally conserved between human and mouse. Among the 1316 circRNA host genes identified in mouse pre-implantation embryos, 835 are also involved in circRNA formation in early human embryos. It was suggested that circRNA host genes in early human embryos may be primarily engaged in the regulation of organelle organization, chromosome organization, cell cycle, and metabolic regulation [23], which is similar to early mouse embryos [71], suggesting that circRNAs may be conserved across species.

The biological function of most detected circRNAs in pre-implantation embryos remains largely unclear. It was suggested that these circRNAs may potentially function as miRNA sponges in the regulation of gene expression during embryo development [72]. An interesting question is why the circRNA number in mouse embryos is significantly less than that in human embryos. Besides some evolutionary factors, it must be noted that there are differences in the technical preparation processes between human [23] and mouse embryo samples [71]. In the study by Dang et al., some human embryos experienced a more complicated process including intracytoplasmic sperm injection, freezing, thawing, and in vitro culture [23], while the mouse embryo samples were freshly isolated [71]. It is unclear if the in vitro manipulation stress triggeres more circRNA expression.

Granulosa cells (GCs) play an important role during oocyte maturation and early embryo development [73, 74]. According to some studies, the gene expression patterns of GCs may be used as potential biomarkers to predict oocyte developmental capacity and consequent assistant reproduction results [75, 76]. Recently, Cheng et al. studied the expression pattern of circRNAs in human GCs during maternal aging [77]. The circRNA microarray technology was used to screen the circRNAs expressed in GCs of patients subjected to in vitro fertilization at a young age (less than 30 years) and an older age (more than 38 years). In older women, the expression levels of 46 circRNAs were up regulated. Meanwhile, the expression levels of 11 circRNAs were down regulated. After validation by reverse transcription PCR (RT-PCR) and adjustment of the effects related to gonadotropin treatment, among these differentially expressed circRNAs, the expression level of circRNA_103827 or circRNA_104816 was found to increase with maternal aging. Moreover, a negative correlation existed between the expression level of both circRNAs and the top quality embryo number. The bioinformatics results demonstrated the involvement of both circRNAs in glucose metabolism, mitotic cell cycle, and ovarian steroidogenesis [77]. The results from the receiver operating characteristic curve analysis revealed a higher sensitivity and specificity when circRNA_103827 or circRNA_104816 was used for prediction of live birth. Therefore, the expression patterns of circRNAs in GCs change with aging. Some specific circRNAs, such as circRNA_103827 or circRNA_104816, may be potential biomarkers for prediction of assistant reproduction outcomes [77].

### Expression of circRNAs in the testis

Spermatogenesis is a complex and precisely-modulated process which is regulated by many testis- or male germ cell-derived genes [72]. During this process, spermatogonial stem cells experience a series of morphological alterations and finally develop into motile sperm [78]. Previous transcriptome studies primarily focus on mRNAs which can be used as a template for protein translation [79]. However, it must be mentioned that the ratio of protein-coding genes in the transcriptome is largely less than that of ncRNAs. Previous studies have indicated that ncRNAs play an important role during mammalian spermatogenesis [80–83]. For example, piwi-interacting RNAs (piRNAs) present in spermatogonia mainly interact with mRNAs and retrotransposons. However, in spermatocytes and spermatids, piRNAs primarily function in intergenic regions [20]. lncRNA has been reported to be engaged in mammalian spermatogenesis [79]. It has been reported that approximately 3000 human lncRNAs are specifically expressed in testis, suggesting that testis is a natural reservoir of lncRNAs [84].

A recent study confirmed the abundant expression of circRNAs in spermatogenic cells. Approximately 15,101 circRNAs were detected in mouse spermatogenic cells [82]. In terms of cell types, 5573, 5596, 6689, 4677, and 7220 circRNAs were expressed in spermatogonial stem cells, primitive type A spermatogonia, preleptotene spermatocytes, pachytene spermatocytes, and round spermatids, respectively. The circRNAs in round spermatids are higher than those in the other cell types [82]. However, the function of the vast amount of circRNAs in testis remains unknown.

In order to reveal the relationship between exon skipping and circRNA biogenesis, the expression patterns of the cytochrome P-450 2C18 gene in human epidermis and the androgen binding protein (ABP) gene in rat testes were investigated [85]. In human epidermis, besides the common mRNAs derived from nine exons, 2C18 circular transcripts skipping exons are also detected. Similarly, in rat testes, a circRNA composed of exons 6 and 7 of the ABP gene was identified. In this circRNA, the donor splice site of exon 7 is linked with the acceptor splice site of exon 6 [85], suggesting that the biogenesis of circRNAs consisting of various exon combinations may be influenced by the generation of mRNAs skipping the exons presented in the testis [85]. Dong et al. explored the expression profiles of circRNAs in human testes and seminal plasma using a high-throughput sequencing technology [20]. 15,996 circRNAs were discovered in the human testes, in which 10,792 circRNAs were novel. Among the detected circRNAs, 14,033 circRNAs can be mapped to 5928 host genes. Among these host genes, 1017 were newly found to generate circRNAs. Most of the host genes are involved in spermatogenesis, motility, and fertilization. The authors also found that these testis-derived circRNAs stably exist in seminal plasma. Furthermore, the circRNAs in seminal plasma are highly stable at room temperature, which may be due to their binding with proteins in seminal plasma. Therefore, it is suggested that the circRNAs in the seminal plasma may be used as novel noninvasive biomarkers for male fertility [20].

### circRNA derived from the Sry gene and sex determination

A study comparing the expression levels of circRNA in brain, liver, heart, lung and testis indicated that circRNA were expressed at high level in testis, only second to that in brain [57], suggesting that circRNAs may have an important role in the testis. A relatively well studied circRNA in the testis is circSry, which is transcribed from the sex-determining region Y (Sry) gene.

In 1990, Sinclair et al. first cloned the Sry gene on the Y chromosome of human sperm [86]. Subsequent studies demonstrated the critically regulatory role of the Sry gene during sex determination [87]. The expression pattern of the Sry gene in mouse testis is developmental stage-dependent [88]. The Sry gene is expressed in the somatic cell lineage of the developing genital ridge between 10.5 and 12.5 days post coitum [87]. During this stage, the Sry gene can be transcribed into a linear mRNA which encodes a protein with a high mobility group (HMG) box. This protein functions as an important transcription factor, regulating sex differentiation during fetal development [89]. On the contrary, in adult testis, the expression of the Sry gene is generally coupled with the first spermatogenesis wave and round spermatid formation. Unlike in the genital ridge, most Sry transcripts in adult testis possess a typically circular structure [88]. It was suggested that long inverted repeats flanking the Sry gene may be responsible for circSry biogenesis [90]. The different transcription pattern of the Sry gene between the genital ridge and adult testis may suggest their specific functions in different developmental stages.

Since circSry possesses an open reading frame and a potential ATG start codon, this circRNA may be capable of protein translation [88]. However, this opinion may be questionable, due to a lack of evidence proving the existence of an interaction between circSry and polysome. Additionally, it is not determined whether circSry can regulate gene expression as a miRNA sponge. In mice, the up-regulation of circSry expression potentially represses the binding of miR-138 with its target mRNAs [27, 36]. According to the report by Hansen et al., biotin-labeled miR-138 has been showed to interact with circSry. Therefore, it is suggested that circSry can function as a miR-138 sponge [27]. However, considering that human circSry has only one miR-138 binding site, the miRNA sponge may be mouse-specific [12, 57]. In addition, the expression pattern of circSry is tissue-specific because circSry cannot be detected in brain, liver, kidney, or spleen [88].

### Expression of circRNAs in placenta

The placenta plays an important role during fetal development, including the regulation of fetal metabolism and nutrition, gas and metabolite exchange, and hormone control [21, 91]. Currently, some studies have found that numerous ncRNAs, such as miRNAs [91, 92] or lncRNAs [93], are expressed in placentas. However, there is limited information related to the expression of circRNAs in placentas.

In a recent study carried out by Qian et al., the Arraystar circRNA microarray technology was used to detect differentially expressed circRNAs in placentas of pregnant women with preeclampsia (PE) [21]. Among 301 differentially expressed circRNAs between the PE placental tissues and the healthy tissues, 143 circRNAs were up-regulated and 158 were down-regulated. Many differentially expressed circRNAs, such as hsa_circRNA_101289, hsa_circRNA_101611, or hsa_circRNA_103285, possess miR-17 binding sites, suggesting that these circRNAs may be involved in the functional regulation of miR-17 in human placentas [21]. miR-17, one of the angiogenesis-associated miRNAs in human placentas, has been reported to highly express in PE placentas [94]. It was supposed that the differentially expressed circRNAs, as miRNA sponges, may competitively bind to miR-17, subsequently sequestering miR-17 and leading to the pathogenesis of PE [21]. In another study by Zhang et al., twelve circRNAs were found to be differentially expressed in blood cells of the PE patients as compared to the healthy women via the circRNA microarray analysis. RT-PCR further confirmed that the expression level of the circ_101222 in blood cells of PE patients was significantly higher than that of healthy women. It was suggested that the combination of circ_101222 with plasma protein factor endoglin may be used as a marker for PE [95].

### circRNAs as biomarkers in the reproductive system

It was recently reported that the expression abundance of circRNAs in highly proliferating cells is generally lower in comparison with cells with lower proliferating capabilities [96, 97]. The expression level of circRNAs in cancerous tissues is less than the corresponding normal tissues, due to a higher proliferation rate in cancerous cells [96]. In ovarian cancer, the abundance of circRNAs in primary ovarian tumors is significantly different from that in metastasis ovarian lesions [98]. It has been reported that the expression level of ciRS-7, as a miR-7 sponge, was significantly up regulated in tumor samples [96]. miR-7 can regulate various oncogenes like Pak1, which is a kinase generally activated by DNA-damaging factors including radiation or etoposide [99]. Therefore, ciRS-7 may be up regulated in cancerous cells to start DNA repair and inhibit apoptosis during exposure to stress [100]. It will be of interest to study if ciRS-7 may be a biomarker for ovary-related cancers.

Recent studies have indicated that thousands of circRNAs are presented in human peripheral whole blood, suggesting that these blood-derived circRNAs may function as potential biomarkers in an easily available bodily fluid [97]. The new finding is from the group of Zhang et al. in which they discovered that circ_101222 in blood cells may function as a biomarker to diagnose PE [95], suggesting that the reproductive status or disease may be assessed via the measurement of the expression pattern of the circRNAs in the blood.

Many procedures in the field of human assisted reproduction, such as freezing and thawing, in vitro maturation, in vitro fertilization, and in vitro culture, can produce great external stress on gametes or embryos. Therefore, cells must adapt to external stresses by adjusting their gene expression patterns. Since circRNAs are abundantly expressed in gametes and embryos, they may be influenced by these procedures.

A typical example is epidermal growth factor (EGF) which is a growth factor that stimulates cell growth, proliferation, and differentiation. EGF has been used for in vitro maturation of mammalian oocytes. During oocyte meiotic maturation from prophase I to metaphase II, luteinizing hormone stimulates the secretion of EGF, which contributes to cumulus cell expansion and oocyte maturation via its receptor EGFR [101]. EGFR is a target of miR-7, therefore its biogenesis is down regulated by this miRNA [102, 103]. Meanwhile, ciRS-7 can sequester miR-7 and block the functional roles of miR-7. Therefore, ciRS-7 may be engaged in the regulation of EGFR function, further influencing oocyte maturation. Additionally, circRNAs are known to be involved in the regulation of cellular response to external stress, such as redox reactions, heat and cold shock, low temperatures, and salt response [66, 104]. It is thus interesting to study how the assisted reproduction process affects the expression patterns of circRNAs and the significance of these gene alterations.

## Conclusions

Although thousands of circRNAs have been detected in ovary, embryo, placenta and testis via high-throughput sequencing technologies, the research on circRNAs in reproductive organs, especially in the ovary, has just begun. The biological functions of these RNAs remain largely unclear. circRNA’s putative functions, such as miRNA sponge, RNA binding protein sponge, and mRNA trap, all add novel layers of regulation network to cell function. More excitingly, the recent findings on confirmation of protein coding function of circRNA, and that m^6^A-driven translation of circRNAs is potentially a general phenomenon, opens a new array of questions to reproductive physiologists: what are the function of these large group of proteins which are previously unknown? Do they function in a similar manner as those derived from mRNA translation? As most circRNAs are derived from 1 to 2 exon canonical genes, translation of them would likely result in a shorter version of mRNA encoding proteins. Would these group of novel isoforms enhance or interfer with canonical protein function? Answering these questions may reveal a new area of understanding in reproductive physiology.

## Abbreviations

ABP: Androgen binding protein
Ack1: Activated cdc42 kinase 1
circMbl: A circRNA derived from the second exon circulation of Mbl pre-mRNA
circRNA: Circular RNA
circSry: A circRNA derived from sex-determined region Y
ciRNA: Circular intronic circRNA
ciRS-7: A circRNA specially bound with miR-7
CLIP-seq: Crosslinking-immunprecipitation and high-throughput sequencing
ecRNA: Exon-derived circRNA
EGF: Epidermal growth factor
EGFR: Epidermal growth factor receptor
EIciRNA: Exon–intron circRNA
EMT: Epithelial-to-mesenchymal transition
FAK: Focal adhesion kinase
fmn: Flavin mononucleotide
GC: Granulosa cell
HMG: High mobility group
HRCR: A heart-related circRNA
IARE: Inverted ALU repeat element
IGF1R: Insulinlike growth factor-1 receptor
IRS-1: Insulin-receptor substrate-1
IRS-2: Insulin-receptor substrate-2
lncRNA: Long non-coding RNA
m^6^A: N^6^-methyladenosine
MBL: Muscleblind
mTOR: Mammalian target of rapamycin
ncRNA: Non-coding RNA
Pak1: p21-activated kinase 1
PE: Preeclampsia
PIK3CD: Phosphatidylinositol-4,5-bisphosphate 3-kinase catalytic subunit delta
piRNA: Piwi-interacting RNA
QKI: Quaking
Raf1: Rapidly accelerated fibrosarcoma 1
RBP: RNA binding protein
RIP-PCR: RNA-binding protein immunoprecipitation- polymerase chain reaction
RIP-qPCR: RNA Immunoprecipitation quantitative PCR
RNase: Ribonuclease
RNA-seq: RNA sequencing
RT-PCR: Reverse transcription PCR
sisRNA: Stable intronic sequence RNA
Sry: Sex-determined region Y
SUPeR-seq: Single-cell universal poly (A)-independent RNA sequencing
U1 snRNP: U1 small nuclear ribonucleoprotein particle
UTR: Untranslated region
